# Optical Design of Textured Thin-Film CIGS Solar Cells with Nearly-Invisible Nanowire Assisted Front Contacts

**DOI:** 10.3390/ma10040392

**Published:** 2017-04-07

**Authors:** Joop van Deelen, Ahmed Omar, Marco Barink

**Affiliations:** 1 TNO/Solliance, High Tech Campus 21, 5656 AE Eindhoven, The Netherlands; a.omar@tue.nl; 2 TNO/Holst, High Tech Campus 31, 5656 AE Eindhoven, The Netherlands; marco.barink@tno.nl

**Keywords:** solar cells, nanogrid, thin-film solar, front contact

## Abstract

The conductivity of transparent front contacts can be improved by patterned metallic nanowires, albeit at the cost of optical loss. The associated optical penalty can be strongly reduced by texturization of the cell stack. Remarkably, the nanowires themselves are not textured and not covered in our design. This was shown by optical modeling where the width of the nanowire, the texture height and the texture period were varied in order to obtain a good insight into the general trends. The optical performance can be improved dramatically as the reflection, which is the largest optical loss, can be reduced by 95% of the original value. The spectra reveal absorption in the Cu(In,Ga)Se_2_ (CIGS) layer of 95% and reflection below 2% over a large part of the spectrum. In essence, a virtually black CIGS cell stack can be achieved for textured cells with a metal nanogrid. Moreover, it turned out that the ratio between the width of the nanowire and the height of the texture is a critical parameter for optical losses.

## 1. Introduction

Transparent conductors are important for the performance of optoelectronic devices. Especially in the field of thin-film solar cells and display technology, high conductivity and high transparency are vital [1,2,3,4]. Traditionally, these transparent conductors are thin layers of doped metal oxide material, the so-called transparent conductive oxides (TCOs) [5,6]. Recently, alternative materials have been under intense research [7,8]. The combination of metallic grids and TCOs was shown to be a good candidate to give a low resistance without compromising the high transmittance [9,10,11,12,13]. Such improvement would be highly beneficial for solar cells [14]. In addition, random silver nanowire networks have received significant interest and large area processing seems to be an available production technology [15,16,17]. A more elegant method of incorporating well defined metal nanostructures could be nanoimprinting [18,19]. Unfortunately, the transparency of these metal nanostructures is often below 80%, depending on the metallic feature size [20,21]. Nevertheless, the unique opto-electronic properties of metallic nanostructures can make them less reflective than expected from their surface coverage [22,23]. Taking this concept one step further, cloaking of metals in metamaterials has been proposed [24,25,26]. 

Recently, a hybrid metal–silicon nanostructure, in which a 16 nm thick gold mesh pattern with 65% surface coverage was deposited on a Si wafer. This was followed by a gold catalyzed etching step, whereby the gold ends up at the bottom of the etched crevice [27]. Such a texturization procedure resulted in a decrease of the initial reflection from 50% for a flat sample (before etching) down to 19% for gold in 330 nm deep trenches after etching. The light was reported to be coupled into the protruding Si nanopillars. This is exciting, because it shows that the combination of nanopattern and nanotexture can reduce the optical loss related to the metal surface coverage. If this could be combined with enhanced incoupling by nanotexturing for thin-film solar cells, then better designs with improved efficiencies are possible. 

However, it seems to be a new development and no reports on such a combination were found for thin-film photovoltaics. Moreover, there are no shared insights into specific optical characteristics with different designs or dimensioning for the metal-in-texture concept, even though large area nanoimprinting fabrication technology has been well developed [28]. Previously, our group has shown design optimization of micro-sized grids as well as nanotexturization of the TCO [4,7,29]. Moreover, the beneficial optical effect of a nanoimprinted anti-reflection coating was experimentally demonstrated on top of a CIGS cell [30,31].

For the present work, optical modeling was used to determine the effect of rectangular nanowires in a triangular multilayer CIGS cell texture. The flat layer stack was used as a reference. The impact of texturization on the absorption of the layers and the total reflection was evaluated by systematical variation of the width of the wire, the period and the height of the texture. Moreover, the absorption spectra were retrieved for all layers, but we focus on the more general trends in optical performance of the thin-film CIGS cells, which were retrieved by calculating the current density from the absorption. The general trends showed surprisingly that the ratio of the width of the wire and the height of the texture is a critical parameter, rather than just the width, height or period. By choosing the right dimensions of the texture and the grid, the reflection can be diminished to levels below 1%. The findings can give guidelines toward promising optical designs.

## 2. Results

**Figure 1:**
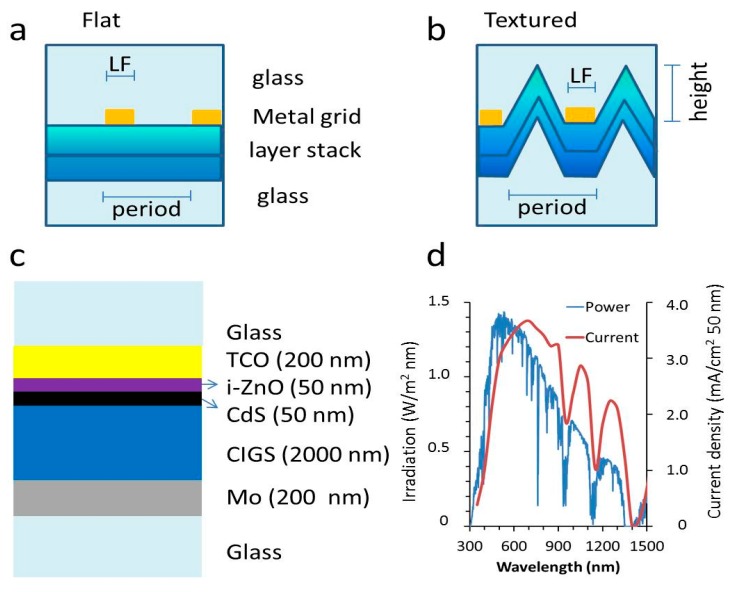
(**a**) Schematic representation of the modeled flat device with the metallic grid on top of the device layer stack. The grid has a width denoted as LF and period, denoted as “period”; (**b**) Schematic representation of the modeled textured device, in which a triangular texture with height (*H*) is introduced; (**c**) Schematic representation of the layer stack without the metallic grid, to detail all the layers. The top and bottom medium were glass in order to represent an encapsulated device; (**d**) Graph displaying the irradiation power density and the correlated current density as a function of the wavelength.

Optical modeling generated the absorption and reflection spectra of the flat layer stack as depicted in Figure 1c, together with the grid lines as sketched in Figure 1a. In addition, various texture dimensions were introduced with the general shape as sketched in Figure 1b, in which the whole thin-film stack followed the texture. For the calculation of the impact of the optical effect in a solar cell device, the current density was calculated using the current spectrum as depicted in Figure 1d.

### 2.1. Spectra of Whole Stack

**Figure 2:**
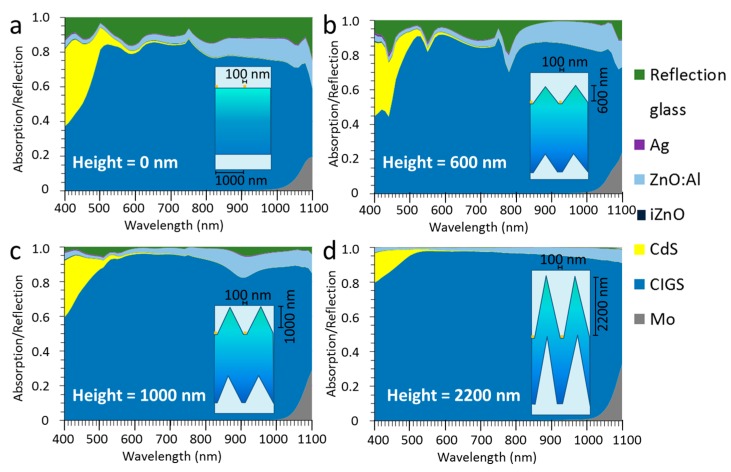
Spectral distributions of the absorption of each material in the stack and the reflection for a nanowire width of 100 nm. The different graphs depict the texture heights of (**a**) 0 nm; (**b**) 600 nm; (**c**) 1000 nm; (**d**) 2200 nm. The period is 1000 nm. The colors in the graphs represent the materials as indicated in the legend, where Ag stands for the absorption in the nanowire material of silver. The reflection is the overall reflection within the glass medium, and therefore represents the internal reflection. The inset shows a sketch of the layout.

The absorption and reflection spectra of the flat layer stack as depicted in Figure 1 with a nanowire width of 100 nm are shown in Figure 2a. The dark blue part in the spectrum is the absorption in the CIGS layer and the other colors represent optical losses. These optical losses are mainly reflection, parasitic absorption in CdS and ZnO:Al. The grey area in the right bottom is the absorption in the Mo layer, underneath the CIGS and this absorption can be interpreted as light that is transmitted through the whole stack and is, therefore, also an optical loss. For flat layers supplemented with a square nanowire (cross section of 100 nm × 100 nm) with a period of 1000 nm, some peaks in the absorption spectra are visible at 490 nm and 750 nm. Here, the reflection is minimized. At a wavelength of 1080 nm, this also seems to be the case. There is 10% of surface coverage by the silver. The total reflection consists of both the reflection of the metal and the internal reflections of the stack. 

When adding a triangular shape with a height of 600 nm for the space between the nanowires, the spectra change as compared to a flat layer configuration (see Figure 2b). The insert in the graphs shows a sketch with the approximate dimensions of wire width, period, texture height and layer thickness to scale. The reflection is reduced, especially for wavelengths above 800 nm, and there are some distinctive peaks in reflection (green surface area in the plots) at 440 nm, 550 nm and 780 nm, which are likely related to resonances of the light with the metal and the TCO. For a metallic grid, Van de Groep et al. find peaks in reflection at similar wavelength ranges and attributes the lower wavelength peaks to localized surface plasmon resonances and the peak at around 800 nm to surface plasmon polariton [20]. They have studied only flat layers, while our case shows that these trends become more pronounced for the textured layer. This leads us to believe that for a textured layer with a metallic grid, the peaks as shown in the figures are the result of various optical phenomena, including surface plasmon resonances, but Mie-like resonance might also play a role, as described by Narasimhan et al., which would be responsible for confining light to the structure [27]. 

With further increase of the texture height to 1000 nm (see Figure 2c), these peaks disappear and the reflection is far below 10%. This shows that with a texture height/period aspect ratio of about 1, the total reflection is significantly lower than the surface coverage of the metal. Apparently, the light is coupled into the structure and circumvents the metal film, as mentioned in [27]. At a height of 2200 nm, the reflection has almost vanished and virtually all the light is absorbed, i.e., a device with this texture would be black, in spite of the “bare” flat silver surface and also the absorption of the silver, which is depicted in purple and is hardly visible in Figure 2d. In other words, the optical losses associated with the silver have disappeared and the silver has become practically invisible. It is thought that on its trajectory towards the metal, much of the light is captured in the solar cell stack before the metal is reached. The light that reaches the metal is mostly reflected, but on its way up, it is captured by the solar cell stack material and does not contribute to external reflection. For heights of 1000 nm and above, the steepness of the solar cell stack also serves as a graded refractive index material, which reduces the reflection of the stack itself (excluding the metal), while the triangle shape enhances incoupling of light inside the triangle. It is thought that this is also an important driver for the disappearance of the resonance peaks. 

The sheet resistance (*R*_sh_) of a metal wire finger pattern can be calculated by using the formula *R*_sh_ = (*ρ*/*h*)·(*a*/*w*), in which *ρ* is the resistivity of the metal (1.59 × 10^8^ Ωm), *h* is the height, *a* is the period and *w* is the width of the metal [32,33]. By using one-third of the bulk resistivity—in order to compensate for material quality and real-life nanowire inhomogeneity—and a nanowire height and width of 100 nm with a period of 1000 nm, we can calculate the sheet resistance for a 10% metal surface coverage to be 4.8 Ω/sq, equivalent to small micron sized grids [34]. For solar cell devices, this is already a suitable value. However, for micron sized metallic grids, a much lower sheet resistance below and around 1 Ω/sq was obtained [7]. In principle, also with a nanogrid 100 nm in height, lower sheet resistances can be reached, if the surface coverage is increased. It is therefore interesting to investigate the optical losses for larger surface coverages by the silver. 

**Figure 3:**
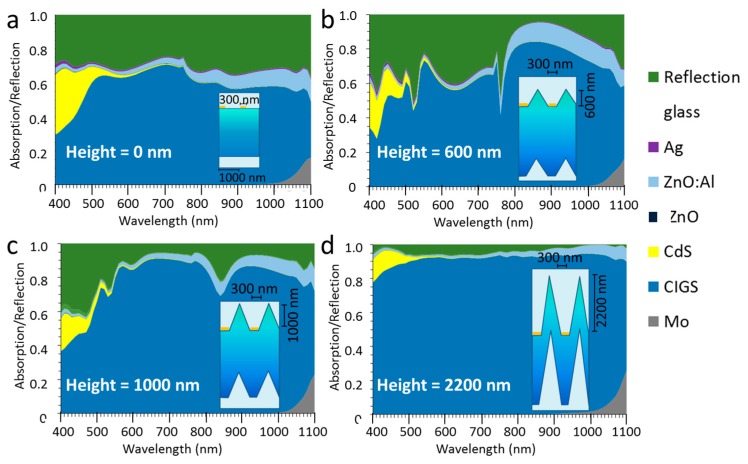
Spectral distribution of the absorption of each material in the stack and the reflection for a nanowire width of 300 nm. The different graphs depict the texture heights of (**a**) 0 nm; (**b**) 600 nm; (**c**) 1000 nm; (**d**) 2200 nm. The period is 1000 nm. The colors in the graphs represent the materials as indicated in the legend, where Ag stands for the nanowire material of silver. The inset shows a sketch of the layout.

The surface coverage was tripled by changing the width of the nanowire to 300 nm with a period of 1000 nm on a flat layer stack. For a flat layer stack, this surface coverage of 30% increases the reflection in an almost linear fashion as shown in Figure 3a. If a triangular texture of 600 nm in height is introduced (Figure 3b), the reflection is reduced for wavelengths above 800 nm. For a texture height of 1000 nm (Figure 3c), the reflection is reduced over a wider wavelength range above 510 nm. If the texture height is 2200 nm, the majority of the absorption is in the CIGS absorber, although there is more reflection than for the 100 nm wide nanowires and the silver wires cannot be considered invisible anymore.

### 2.2. Trends of CIGS and Reflection Spectra

**Figure 4:**
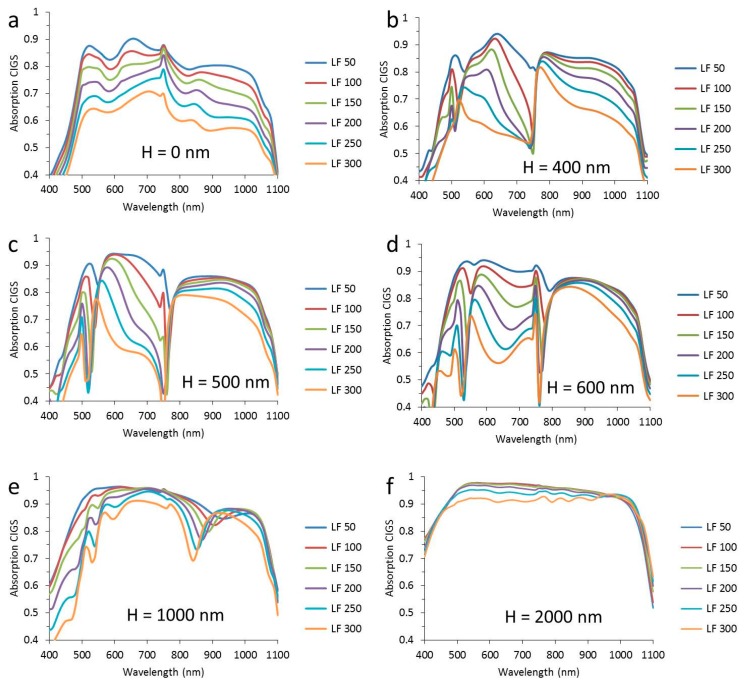
The absorption in the CIGS layer as a function of the wavelength for a period of 1000 nm and a texture height (H) of (**a**) 0 nm; (**b**) 400 nm; (**c**) 500 nm; (**d**) 600 nm; (**e**) 1000 nm; (**f**) 2000 nm. The width of the nanowire (LF) varies between 50 nm and 300 nm, as indicated in the legend.

**Figure 5:**
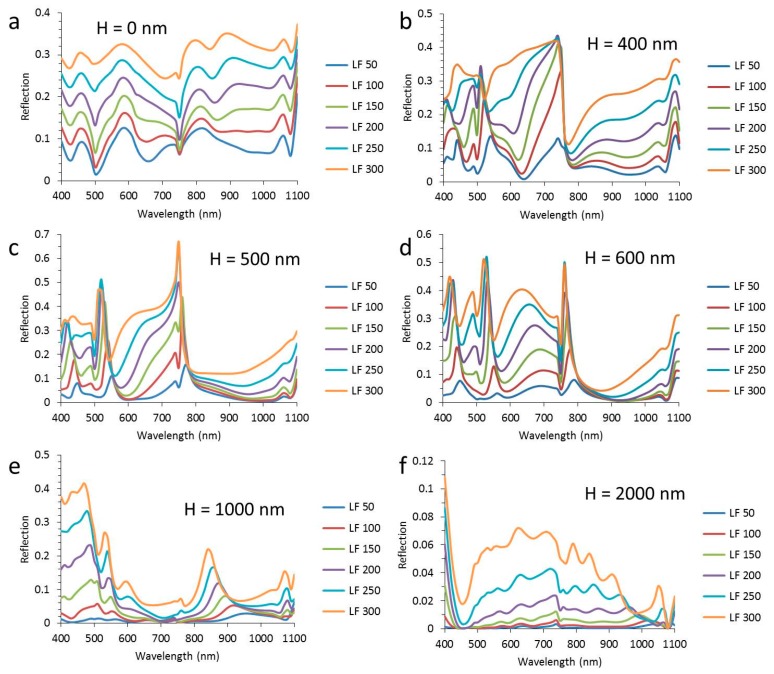
The total reflection as a function of the wavelength for a period of 1000 nm and a texture height (*H*) of (**a**) 0 nm; (**b**) 400 nm; (**c**) 500 nm; (**d**) 600 nm; (**e**) 1000 nm; (**f**) 2000 nm. The width of the nanowire (LF) varies between 50 nm and 300 nm, as indicated in the legend.

In order to get a more detailed picture of the wavelength distribution of CIGS absorption and total reflection, these spectra are shown with the whole range of wire widths from 50 nm to 300 nm in each graph in Figure 4 and Figure 5. In Figure 4, the absorption spectra of the CIGS are shown for six different texture heights from 0 nm to 2000 nm as indicated in the Figure 4a–f. The presented heights have been selected based on the most illustrative changes in the spectra. 

For flat layers, the absorption of the CIGS shows a decreasing trend with increasing nanowire width. This decrease is more or less evenly distributed over the whole wavelength range, except at 750 nm, where there is a peak in absorption, which is almost constant up to a nanowire width of 150 nm. The absorption in CIGS decreases with nanowire width, because the wider nanowires induce higher reflections (see Figure 5a). For a texture height of 400 nm, there is a dramatic change in the trend of the spectral distribution with nanowire width. Instead of a peak (observed for flat layer stacks), there is a dip at 750 nm. Even for a nanowire width of 100 nm (i.e., a surface coverage of 10%) this dip gives a CIGS absorption of only 50%, i.e., the optical loss is larger at a wavelength of 750 nm than “expected” from the surface coverage. Further increase of the nanowire width does not reduce the absorption at 750 nm, but increases the wavelength range, turning the dip into a broad valley. For a texture height of 500 nm, a similar trend is visible. At a texture height of 600 nm, the CIGS absorption above a wavelength of 800 nm is high and is hardly influenced by the nanowire width, while below 750 nm, the width has a strong impact. For a texture height of 1000 nm (Figure 4e), the impact of the nanowire width is relatively small except for wavelengths below 550 nm and around 850 nm. For a texture height of 2000 nm, there is little impact of the nanowire width below widths of 250 nm and the CIGS absorption is about 0.95 over a large wavelength range. 

Most of the trends seen in the CIGS absorption spectra are directly linked to the trends in the reflection shown in Figure 5. For instance, there is a peak in absorption in the CIGS at a wavelength of 750 nm for flat layers, while there is a dip in reflection. Also, for the textured configurations, a good correlation between the trends in CIGS absorption and reflection is seen. The dip at 750 nm for flat layers becomes a peak for textured layers. For texture heights up to 500 nm, the reflection ranges from practically 0 to almost 0.7, depending on the wavelength and the surface coverage of the metal. For a texture height of 1000 nm, the trend in the reflection spectra is different than for lower textures as Figure 5e shows little change in reflection for wavelengths between 600 nm and 800 m, in contrast to all other heights. The texture height of 2000 nm induces a drop in reflection to a fraction of the metal surface coverage (note that the scale of the *y*-axis is up to 0.12). Moreover, the reflection is more evenly spread over the wavelength range compared to lower texture heights.

In summary, the absorption and reflection spectra reveal how the spectral distribution of CIGS absorption and reflection changes with texture height and nanowire width. Most notably, there is a sort of threshold wavelength at 750 nm above which the reflection is low. For high textures, the reflection is low over the entire spectrum between 400 nm and 1100 nm.

Qualitatively, the trends with increasing nanowire width are in agreement with van de Groep et al. [15], who also observe a deepening and widening of the reflection at low wavelengths. However, there are also distinctive differences between their results on flat and transparent substrates and our not transparent textured substrates, and for this reason, we cannot unambiguously explain our observations with just localized surface plasmon and surface plasmon polaritons and a more detailed study is needed to explain these results. However, the main objective of this contribution is to show the general trends and focus on its practical effect in devices. 

### 2.3. Current Densities of CIGS and Optical Loss

**Figure 6:**
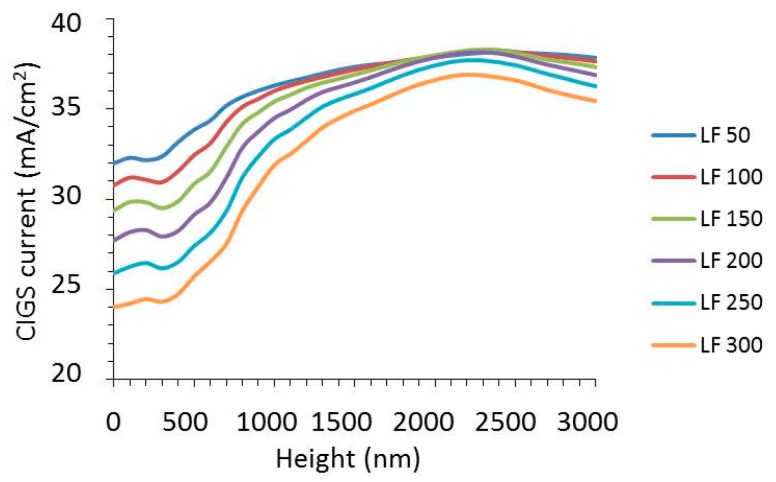
The CIGS absorption of a textured device expressed in current density with metal nanowires as a function of the texture height for the different nanowire widths (LF) between 50 nm and 300 nm as indicated in the legend.

The optical behavior can be translated into device performance and Figure 6 shows the CIGS current density as obtained from absorption spectra as a function of the texture height for various nanowire widths (LF) between the triangles of the texture. The absorption in CIGS is displayed in mA/cm^2^. This value is obtained by multiplying the absorption spectra with the current density spectrum, which was calculated from the AM1.5G spectrum (see Figure 1d). For low texture heights, cell stacks supplied with nanowires on the flat surface areas clearly show the impact of reflection related to the nanowires, which is equivalent to their surface coverage (Figure 6). However, as the height of the texture increases above 1000 nm, the impact of the nanowires diminishes. For wider nanowires, some optical loss remains present even for large texture heights.

**Figure 7:**
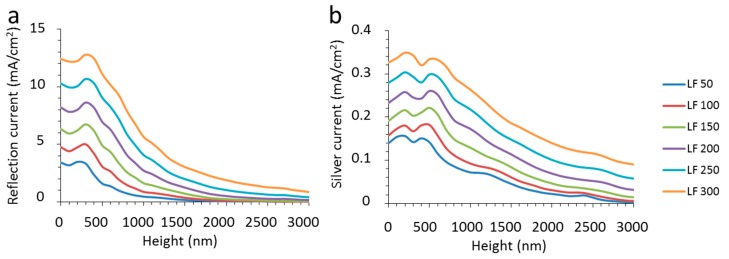
(**a**) The total reflection expressed in current density as derived from the spectra as described in the text as a function of the texture height for the different nanowire widths (LF) between 50 nm and 300 nm as indicated in the legend; (**b**) the absorption of the silver nanowires expressed in current density as derived from the spectra as described in the text as a function of the texture height for the different nanowire widths (LF) between 50 nm and 300 nm as indicated in the legend.

The optical losses of the total reflection and the absorption by the silver are shown in Figure 7. Similar to the absorption of the CIGS, the values are shown in the unit of mA/cm^2^. In spite of the distinctive differences between the spectra as shown in Section 2.2, there is a clear consistency in trends for the increase in reflection with surface coverage. The reflection remains relatively constant with height, before it reaches a small maximum at a texture height of about 300 nm. With further increase of the texture height beyond this threshold, the reflection drops significantly. At texture heights above 2000 nm, there is only a very small amount of reflection and the textured device should appear essentially black, as mentioned above. This shows that the substantial loss of reflection for flat devices with partially metallized front surfaces can be diminished by texturization. The current loss caused by the absorption of the silver is much lower than the reflection, but also displays similar trends. In general, there is a clear dependence on both texture height and nanowire width, but from these figures it is unclear whether there is a real relationship between the two.

**Figure 8:**
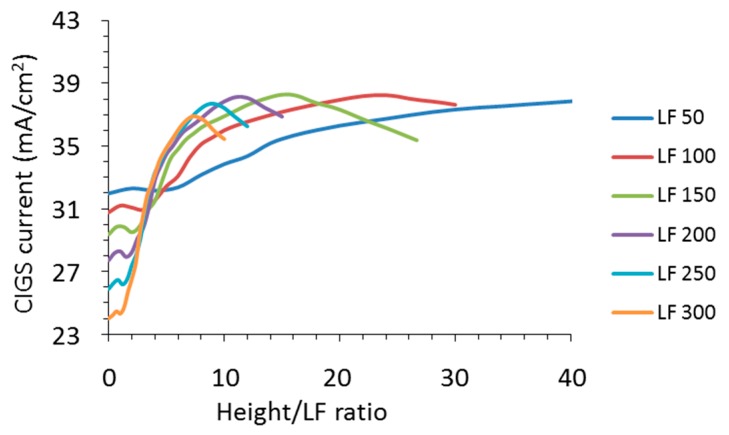
The CIGS absorption of a textured device expressed in current density with metal nanowires as a function of the ratio of texture height and nanowire width (height/LF ratio) for the different nanowire widths between 50 nm and 300 nm as indicated in the legend.

**Figure 9:**
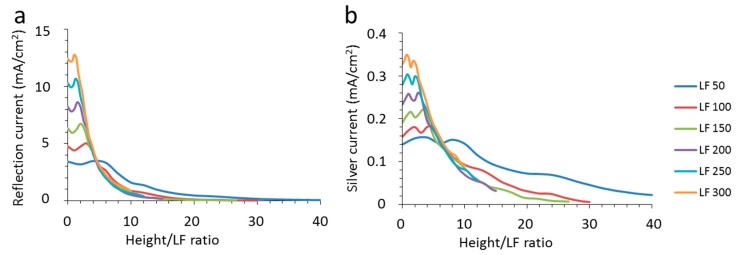
(**a**) The total reflection expressed in current density as derived from the spectra as described in the text as a function of the ratio of texture height and nanowire width (height/LF ratio) for the different nanowire widths (LF) between 50 nm and 300 nm as indicated in the legend; (**b**) the absorption of the silver nanowires expressed in current density as derived from the spectra as described in the text as a function of the ratio of texture height and nanowire width for the different nanowire widths between 50 nm and 300 nm as indicated in the legend.

The relation between the optical characteristics and the geometries of grid and texture suggest that increasing texture height and decreasing grid width reduce the optical losses. For improved insight into the interrelation of these trends, the absorption of CIGS and silver and the total reflection is displayed against the ratio of the texture height and the nanowire width (height/LF ratio on the *x*-axis) in Figure 8 and Figure 9. If we use this *x*-axis, the absorption values of CIGS show distinctive overlap along a single line in the graph. Also, a maximum is observed for each line, which shifts with the height/LF ratio for different nanowire widths. These maxima represent the maxima observed for a texture height of 2200 nm, which is the same for all nanowire widths. 

The overlap is even more visible when the reflection is put as a function of the height/LF ratio (see Figure 9a). Apparently, it is this ratio that is the determining factor rather than the texture height or the width of the nanowire. Moreover, a height/LF ratio of 10 results in a strongly diminished reflection. In other words, the required texture height needed for reduced reflection follows directly from the chosen width of the nanowire in case of a triangular texture. Moreover, there seems to be a kind of threshold of the height/LF ratio before the reflection starts to reduce, which is dependent on the nanowire width (LF). As soon as this plateau reaches the line of the overlap, it follows this “overlap” line. For an LF of 300 nm, the reflection drops from a height/LF ratio of 2, whereas, for an LF of 100 nm, a height/LF ratio of 4 is needed before the reflection starts to decrease. 

The absorption of the nanowire shows this trend as well (see Figure 9b). These trends give more insights into the boundary conditions of cloaking nanowires. Previously, it was reported that the reflection of nanowires could be reduced by about 60% by putting them at the bottom of rectangular trenches of silicon [27] and here we show a general design rule for a CIGS cell stack and reach theoretical values of more than 95% reduction of reflection for certain wavelength ranges. It is envisioned that the specific reflection for a certain height/LF ratio also depends on the materials used and the texture shape, but that falls outside the scope of this study, which focuses on the trends of nanowires with a CIGS cell stack and triangular texture.

## 3. Methods

Modeling is a good way to evaluate a wide array of parameters and to determine the impact of the design of devices and equipment on their functionality [30,35]. Optical modeling is powerful for gaining insight into what would otherwise be invisible or impossible to measure, especially in the nanosized domain [23,36,37]. We used FEM-based wave-optics modeling with the Comsol Multiphysics package (Comsol AB, Stockholm, Sweden). It solves the Maxwell equations and has a relatively easy way to adjust the geometry. The semi-automated geometry input in the solver and the processing of the output was done with a Matlab (Mathworks©, Natick, MA, USA) interface. Because of the large amount of texture dimensions, 2D modeling was applied with the magnetic field perpendicular to the simulation plane. 

**Figure 10:**
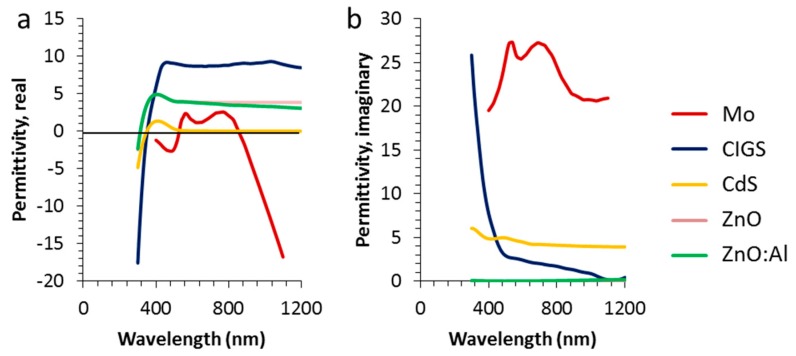
Real (**a**) and imaginary (**b**) permittivities used for the modeling [38,39,40,41,42].

Device architectures with and without texture were evaluated. Schematic representations of the flat and textured designs are shown in Figure 1. The flat layer case shows a rectangular metallic grid with a height of 100 nm and a width “LF”. The solar cell layer stack is shown in more detail in Figure 1c. Behind glass, a front contact TCO is followed by a CdS buffer layer. The CIGS absorber is taken as a homogeneous layer and the back contact is a molybdenum layer on a glass substrate. The top medium is glass and therefore, the 4% reflection associated with the glass/air interface is not included in these calculations. This way, the impact of the texture and the metal can be assessed more clearly without this 4% offset. The optical data were retrieved from the literature and shown in Figure 10 [38,39,40,41,42]. The width of the nanowire LF was varied between 25 nm (5% surface coverage) and 150 nm (30% surface coverage). In case of the textured cells, the cell area underneath the metal nanowire with width LF was kept flat (i.e., horizontal) and the triangular features were put between. Thus, the base of the triangle is set to period minus LF. The period was set to 1000 nm. The height of the texture was varied from 0 up to 3000 nm and it was assumed that all the layers follow this texture, i.e., the vertical layer thickness of each particular layer is constant over the whole surface. For the texture, a 2D approach was chosen in order to accommodate a large variety of geometries with a reasonable calculating time, while still retaining sufficient details of optical phenomena.

For device applications, the most important aspects are the practical consequences of the optical characteristics induced by the different texture sizes. Therefore, we focus on the net optical effects and present them in spectral distributions, with special emphasis on absorption in the CIGS layer and reflection. These are translated into the relevant cell parameter of current density (mA/cm^2^) by multiplication of the absorption spectrum of the CIGS layer and the photon density spectrum (shown in Figure 1d). This was done to evaluate the impact of the design in more practical terms in contrast to reports that show spectacular gains at specific wavelengths but have limited practical impact on device performance.

## 4. Conclusions

Metallic nanowires could boost the conductivity of the front conductor in thin-film photovoltaics (PV). However, normally, this comes at an optical penalty. The optical issue can be solved by introducing a texture. The optical characteristics of a triangular textured thin-film CIGS cell stack with a rectangular metallic grid were modeled. The width of the nanowire, the texture height and the texture period were varied in order to obtain a good insight into the general trends. The spectra reveal CIGS absorption of 95% and reflection below 2% over large parts of the spectrum, even though the metal surface is “exposed” and remains flat in the presented design. Optimized optical performance was obtained for a texture height of 2200 nm of a period of 1000 nm. Moreover, it turned out that the ratio of the texture height and the width of the nanowire is an all-important parameter, determining the optical losses related to the nanowire. If the texture height/nanowire width ratio is 10, the reflection is extremely low. The trends observed in this modeling can serve as general design rules for device optimization. 

## References

[1] Brecl K., Topič M. (2008). Simulation of losses in thin-film silicon modules for different configurations and front contacts. Prog. Photovolt. Res. Appl..

[2] Keller J., Lindahl J., Edoff M., Stolt L., Törndahl T. (2016). Potential gain in photocurrent generation for Cu(In,Ga)Se_2_ solar cells by using In_2_O_3_ as a transparent conductive oxide layer. Prog. Photovolt. Res. Appl..

[3] Van Deelen J., Tezsevin Y., Barink M. (2016). Multi-material front contact for 19% thin film solar cells. Materials.

[4] Blakers A.W. (1992). Shading losses of solar-cell metal grids. J. Appl. Phys..

[5] Van Deelen J., Barink M., Klerk L., Voorthuijzen W.P., Hovestad A. (2015). Efficiency loss prevention in monolithically integrated thin film solar cells by improved front contact. Prog. Photovolt. Res. Appl..

[6] Morales-Masis M., de Nicolas S.M., Holosky J., de Wolf S., Ballif C. (2015). Low-temperature high-mobility amorphous IZO for silicon heterojunction solar cells. IEEE J. Photovolt..

[7] Illiberi A., Kniknie B., van Deelen J., Steijvers H.L.A.H., Habets D., Simons P.J.P.M., Janssen A.C., Beckers E.H.A. (2011). Industrial high-rate (~14 nm/s) deposition of low resistive and transparent ZnO*_x_*:Al films on glass. Sol. Energy Mater. Sol. Cells.

[8] Ward J.S., Duda A., Friedman D.J., Geisz J., McMahon W., Young M. (2015). High aspect ratio electrodeposited Ni/Au contacts for GaAs-based III-V concentrator solar cells. Prog. Photovolt. Res. Appl..

[9] Van Deelen J., Klerk L.A., Barink M., Rendering H., Voorthuizen P., Hovestad A. (2014). Improvement strategy of transparent conductive materials using metallic grids: Modeling and experiments. Thin Solid Films.

[10] Van Deelen J., Frijters C. (2017). Metallized front contact for higher efficiency CIGS cells. Sol. Energy.

[11] Hsu P.C., Wang S., Wu H., Narasimhan V.K., Kong D., Lee H.R., Cui Y. (2013). Performance enhancement of metal nanowire transparent conducting electrodes by mesoscale metal wires. Nat. Commun..

[12] Kik P.G. (2014). Catoptric electrodes: Transparent metal electrodes using shaped surfaces. Opt. Lett..

[13] Saive R., Bukowsky C.R., Yalamanchili S., Boccard M., Saenz T., Borsuk A.M., Holman Z., Atwater H.A. Effectively transparent contacts (ETCs) for solar cells. Proceedings of the 2016 IEEE 43th Photovoltaic Specialists Conference (PVSC).

[14] Van Deelen J., Klerk L., Barink M. (2014). Optimized grid design for thin film solar panels. Sol. Energy.

[15] De S., Higgins T.M., Lyons P.E., Doherty E.M., Nirmalraj P.N., Blau W.J., Boland J.J., Coleman J.N. (2009). Silver nanowire networks as flexible, transparent, conducting films: Extremely high DC to optical conductivity ratios. ACS Nano.

[16] Kwan Y.C.G., Le Q.L., Huan C.H.A. (2016). Time to failure modeling of silver nanowire transparent conducting electrodes and effects of a reduced graphene oxide over layer. Sol. Energy Mater. Sol. Cells.

[17] Lee J.Y., Connor S.T., Cui Y., Peumans P. (2008). Solution-processed metal nanowire mesh transparent electrodes. Nano Lett..

[18] Kang M.G., Park H.J., Ahn S.H., Guo L.J. (2010). Transparent Cu nanowire mesh electrode on flexible substrates fabricated by transfer printing and its application in organic solar cells. Sol. Energy Mater. Sol. Cells.

[19] Van de Groep J., Gupta D., Verschuuren M.A., Wienk M.M., Janssen R.A.J., Polman A. (2015). Large-area soft-imprinted nanowire networks as light trapping transparent conductor. Sci. Rep..

[20] Van de Groep J., Spinelli P., Polman A. (2012). Transparent conducting silver nanowire networks. Nano Lett..

[21] Kang M.G., Guo L.J. (2007). Nanoimprinted semitransparent metal electrodes and their application in organic light-emitting diodes. Adv. Mater..

[22] Afshinmanesh F., Curto A.G., Milaninia K.M., van Hulst N.F., Brongersma M.L. (2014). Transparent metallic fractal electrodes for semiconductor devices. Nano Lett..

[23] Nakanishi T., Tsustumi E., Masunaga K., Fujimoto A., Asakawa K. (2011). Transparent aluminum nanomesh electrode fabricated by nanopatterning using self-assembled nanoparticles. Appl. Phys. Express.

[24] Landy N., Smith D.R. (2013). A full-parameter unidirectional metamaterial cloak for microwaves. Nat. Mater..

[25] Schumann M.F., Wiesendanger S., Goldschmidt J.C., Bläsi B., Bittkau K., Paetzhold U.W., Sprafke A., Wehrspohn R.B., Rockstuhl C., Wegener M. (2015). Cloaked contact grids on solar cells by coordinate transformations: Designs and prototypes. Optica.

[26] Chen F.H., Pathreeker S., Kaur J., Hosein A.D. (2016). Increasing light capture in silicon solar cells with encapsulants incorporating air prisms to reduce metallic contact losses. Opt. Express.

[27] Narasimhan V.K., Hymel T.M., Lai R.A., Cui Y. (2015). Hybrid metal-semiconductor nanostructure for ultrahigh optical absorption and low electrical resistance at optoelectronic interfaces. ACS Nano.

[28] John J., Tang Y.Y., Rothstein J.P., Watkins J.J., Carter K.R. (2013). Large-area; continuous roll-to-roll nanoimprinting with PFPE composite molds. Nanotechnology.

[29] Xu M., Wachters A.J.H., van Deelen J., Mourad M.C.D., Buskens P.J.P. (2014). A study on the optics of copper indium gallium (di)selenide (CIGS) solar cells with ultra-thin absorber layers. Opt. Express.

[30] Burghoorn M., Kniknie B., van Deelen J., Xu M., Vroon Z., van Ee R., van de Belt R., Buskens P. (2014). Improving the efficiency of copper indium gallium (Di-)selenide (CIGS) solar cells through integration of a moth-eye textured resist with a refractive index similar to aluminum doped zinc oxide. AIP Adv..

[31] Buskens P., Burghoorn M., Mourad M.C.D., Vroon Z. (2016). Antireflective coatings for glass and transparent polymers. Langmuir.

[32] Catrysse P.B., Fan S. (2010). Nanopatterned metallic films for use as transparent conductive electrodes in optoelectronic devices. Nano Lett..

[33] Kuang P., Park J.-M., Leung W., Mahadevapuram R.C., Nalwa K.S., Kim T.-G., Chaudhary S., Ho K.-M., Constant K. (2011). A new architecture for transparent electrodes: Relieving the trade-off between electrical conductivity and optical transmittance. Adv. Mater..

[34] Saive R., Borsuk A.M., Emmer H.S., Bukowsky C.R., Lloyd J.V., Yalamanchili S., Atwater H.A. (2011). Effectively Transparent Front Contacts for Optoelectronic Devices. Adv. Opt. Mater..

[35] Van Deelen J., Illiberi A., Kniknie B., Steijvers H., Lankhorst A., Simons P. (2013). APCVD of ZnO:Al, insight and control by modeling. Surf. Coat. Technol..

[36] Gao T., Leu P.W. (2013). The role of propagating modes in silver nanowire arrays for transparent electrodes. Opt. Express.

[37] Brongersma M.L., Cui Y., Fan S. (2014). Light management for photovoltaics using high-index nanostructures. Nat. Mater..

[38] Jarzembowski J., Maiberg M., Obereigner F., Kaufmann K., Krause S., Scheer R. (2015). Optical and electrical characterization of Cu(In,Ga)Se_2_ thin film solar cells with varied absorber layer thickness. Thin Solid Films.

[39] Lundberg O., Bodegard M., Malmström J., Stolt L. (2003). Influence of the Cu(In,Ga)Se_2_ Thickness and Ga Grading on Solar Cell Performance. Prog. Photovolt. Res. Appl..

[40] Fujiwara H., Kondo M. (2005). Effects of carrier concentration on the dielectric function of ZnO:Ga and In_2_O_3_:Sn studied by spectroscopic ellipsometry: Analysis of free-carrier and band-edge absorption. Phys. Rev. B.

[41] Schultz L.G., Tangherlini F.R. (1954). Optical Constants of Silver, Gold, Copper, and Aluminum. I. The Absorption Coefficient k. J. Opt. Soc. Am..

[42] Schultz L.G., Tangherlini F.R. (1954). Optical Constants of Silver, Gold, Copper, and Aluminum. II. The Index of Refraction n. J. Opt. Soc. Am..

